# Improving palliative care with machine learning and routine data: a rapid review

**DOI:** 10.12688/hrbopenres.12923.2

**Published:** 2019-08-12

**Authors:** Virginia Storick, Aoife O’Herlihy, Sarah Abdelhafeez, Rakesh Ahmed, Peter May

**Affiliations:** 1 School of Medicine, Trinity College Dublin, Dublin, D02, Ireland; 2 Centre for Health Policy and Management, Trinity College Dublin, Dublin, D02, Ireland; 3 The Irish Longitudinal study on Ageing, Trinity College Dublin, Dublin, D02, Ireland

**Keywords:** Machine learning, artificial intelligence, palliative care, terminal care, multimorbidity, quality of life, costs, decision-making

## Abstract

**Introduction: **Improving palliative care is a priority worldwide as this population experiences poor outcomes and accounts disproportionately for costs. In clinical practice, physician judgement is the core method of identifying palliative care needs but has important limitations. Machine learning (ML) is a subset of artificial intelligence advancing capacity to identify patterns and make predictions using large datasets.  ML has the potential to improve clinical decision-making and policy design, but there has been no systematic assembly of current evidence.

**Methods: **We conducted a rapid review, searching systematically seven databases from inception to December 31st, 2018: EMBASE, MEDLINE, Cochrane Library, PsycINFO, WOS, SCOPUS and ECONLIT.  We included peer-reviewed studies that used ML approaches on routine data to improve palliative care for adults.  Our specified outcomes were survival, quality of life (QoL), place of death, costs, and receipt of high-intensity treatment near end of life.  We did not search grey literature.

**Results:** The database search identified 426 citations. We discarded 162 duplicates and screened 264 unique title/abstracts, of which 22 were forwarded for full text review.  Three papers were included, 18 papers were excluded and one full text was sought but unobtainable.  One paper predicted six-month mortality, one paper predicted 12-month mortality and one paper cross-referenced predicted 12-month mortality with healthcare spending.  ML-informed models outperformed logistic regression in predicting mortality where data inputs were relatively strong, but those using only basic administrative data had limited benefit from ML.  Identifying poor prognosis does not appear effective in tackling high costs associated with serious illness.

**Conclusion: **While ML can in principle help to identify those at risk of adverse outcomes and inappropriate treatment, applications to policy and practice are formative.  Future research must not only expand scope to other outcomes and longer timeframes, but also engage with individual preferences and ethical challenges.

## Introduction

### Background

Improving care for people with serious and complex medical illness is a health system priority worldwide. Between 2016 and 2060 there will be an estimated 87% increase globally in the number of deaths that occur following serious health-related suffering, with low-income countries experiencing the largest proportional increases ^1^. Health systems originally configured to provide acute, episodic treatment often provide poor-value care to complex multimorbid populations ^2^.

Palliative care is an approach “that improves the quality of life (QOL) of patients and their families facing the problems associated with life-threatening illness, through the prevention and relief of suffering by means of early identification and impeccable assessment and treatment of pain and other problems, physical, psychosocial and spiritual” ^3^. Studies suggest that palliative care improves outcomes and reduces health care costs for people with serious medical illness, although significant gaps in the evidence base persist ^4^. Insufficient palliative care capacity is reported even among those nations whose services perform strongly on international rankings ^5, 6^, and need will only grow given demographic ageing ^7^.

In clinical practice, physician judgement remains the *de facto* method of identifying palliative care needs and predicting adverse outcomes including mortality ^8^. However, studies have repeatedly shown that clinicians tend to make imprecise and overly optimistic predictions of survival in metastatic cancer, where prognosis is most accurate of major terminal illnesses ^9– 11^. No subgroup of clinicians are proven to be more accurate than others in late-stage predictions ^8^. 

For research and policy, primary research studies are rare due to ethical and practical issues, increasing reliance on the use of routine data ^4, 12^. Evaluations of programmes and interventions employing traditional analytic approaches encounter challenges in missing data and unobserved confounding ^13^.

Health care is entering an era of ‘big data’ in which researchers and providers will have access to unprecedented levels of information on patients ^14^. Machine learning (ML) is a subset of artificial intelligence that is rapidly advancing capacity to identify patterns and make predictions using large datasets ^15^. In contrast to traditional analytic methods, where the analyst specifies data inputs according to hypotheses and/or conceptual models, ML approaches leverage computing power to identify patterns in available data and can make inferences without explicit user instruction ^16^. These have a well-documented potential to improve clinical decision-making by analysing electronic health records and other routinely collected health data that are commonly large and “dirty”, i.e. erroneous and incomplete ^17^, although significant data missingness may be still be a source of bias ^18^.

### Rationale and aim

ML approaches to improving decision-making may be strongly appropriate for palliative care. First, patient care is complex with high illness burden and management challenges including polypharmacy ^19^. ML tools may be effective at identifying patterns in data on complex patients, for example denoting risk of functional decline or mortality, that are not obvious to healthcare professionals with limited time and analytic expertise to interpret available data. Second, the volume of projected palliative care need means that a minority of patients will receive care from palliative care specialists ^7, 20^. Tools that can aid non-specialists to identify need and provide appropriate care are essential to avoiding population health crises in an era of demographic ageing ^21^. While multiple face-to-face clinical assessment tools exist for this purpose, meeting challenges of scale requires improved use of routinely collected data. Third, in the context of reliance on routine data ^4, 12, 13^. Since ‘big’ data are largely routine data, optimising analytic approaches to these may be particularly impactful in palliative care compared to fields where randomised trials and large primary data collection are more feasible and commonplace.

Multiple recent editorials in topic journals reflect the growing interest in principle in using big data and ML to improve palliative care ^22– 25^. Less has been written on the empirical evidence of these applications. In this context we conducted a rapid review of research studies using ML to improve palliative care. By identifying and organising this evidence for the first time, we anticipated that our findings could inform ongoing and future efforts in this field. 

## Methods

### Eligibility criteria

We included studies meeting the following PICOS (Population, Intervention, Comparison, Outcomes and Study design) criteria.

***Types of participants*.** Studies that reported on adults (≥ 18 years). We specified this criterion as children are a distinct and functionally different population. Studies that reported adults and children separately were eligible, but only results for adults would be considered; studies that pooled adults and children in one sample were excluded ^26^. We sought studies seeking to improve palliative care: care that “improves the quality of life of patients and their families facing the problems associated with a life-threatening illness” ^3^. Studies not addressing palliative care, or evaluating a specific discrete treatment (e.g. stent, chemotherapy) were excluded.

***Types of outcomes*.** We specified three domains of interest: patient outcomes (survival; QoL); caregiver outcomes (survival; QoL); and economic outcomes (costs; receipt of cost-(in)effective treatment, e.g. high-intensity treatment at end of life). We specified these as established measures for quality in palliative and EOL care, and so of particular interest to practitioners and policymakers ^27^.

***Types of studies and reports*.** We included the following types of studies: those that used routinely collected data with ML approaches to improve palliative care, connecting our outcomes of interest with patients’ characteristics before or at diagnosis. Any prospective study meeting the other criteria was therefore eligible; retrospective studies were eligible provided that the analysis was conducted counting *forwards*, i.e. defining samples and treatments at baseline. Studies that counted backwards, examining samples defined by characteristics at death or according to outcome, were excluded for three reasons. First, participant selection at death biases substantially derived results by distorting timeframe of analysis ^28^. Second, mortality is an outcome and many characteristics at death are outcomes; they are not independent of treatment choices but instead endogenous and therefore inappropriate as an eligibility criteria for evaluating the treatment ^29^. Third, treatment effect estimates from decedent cohort studies are in practice evidence that “treatment *t* should be provided to population *p*”, where *p* is partly defined by imminent death ^28, 29^. They are therefore only useful under scenarios of very good prognostic accuracy, and this is seldom the case in the real world ^8– 11^.

This was a rapid review of published literature. Rapid reviews are a well-established methodology for gathering evidence in a limited timeframe, provided that search methods are transparent and reproducible, clear inclusion criteria are applied, and a rigorous if time-limited appraisal is performed ^30, 31^. Only peer-reviewed papers in academic journals were considered eligible. Selection of relevant papers was restricted to English-language publications only. We excluded conference abstracts and book chapters returned by the database search, and we did not search grey literature.

### Information sources

***Database search*.** The following electronic databases were searched:

- EMBASE

- MEDLINE

- Cochrane Library

- PsycINFO

- Web of Science (WOS)

- SCOPUS

- ECONLIT

Searches were conducted on February 14 ^th^, 2019. All databases were searched from their inception dates to December 31 ^st^, 2018.

A full search strategy is provided in Table 1. Search terms for all seven databases are provided as extended data ^32^.

**Table 1.** Example search strategy, EMBASE.

#	Limiters
1	'palliative therapy'/exp OR 'terminal care'/exp OR 'terminally ill patient'/exp OR 'hospice'/exp
2	Palliat*:ti,ab
3	(‘terminal illness’ OR ‘end of life’ OR ‘end-of-life’ OR ‘end-stage disease’ OR ‘last year of life’):ab,ti
4	#1 OR #2 OR #3
5	‘machine learning’/exp
6	(‘data mining’ OR ‘artificial intelligence’ OR ‘machine learning’ OR ‘deep learning’ OR ‘neural networks’):ti,ab
7	#5 OR #6
8	#4 AND #7
9	Limiters on #8: To end of 2018; articles and reviews and articles in press only. NOT conference proceedings or a book chapters.

### Study selection

***Screening of titles and abstracts*.** Two reviewers (any two authors) independently screened each title and abstract of retrieved citations based on the eligibility criteria. Subsequently, conflicts were resolved on the basis of consensus. The online reviewer tool Covidence was used to manage the screening and selection process.

***Screening of full-text reports*.** The second phase of screening involved downloading and assessing the full-text papers for all of the citations retained from the first phase of screening and applying the eligibility criteria. Two independent reviewers (any two of VS, AOH, SA, RA) screened each full-text paper.

***Hand searching*.** One author (PM) searched the ferences of all included papers by hand, applying the same criteria.

***Data extraction*.** We extracted study design features to a standardised template: setting (country, year, health care environment), aim, principle methods, data sources and sample size. These are presented in Table 2.

**Table 2.** Key characteristics of included studies.

Lead author (year)	Setting	Aim	Principle methods	Data and sample	Key Results
Einav (2018) ^33^	US: random sample of Medicare fee-for- service beneficiaries in 2008	To analyse healthcare spending by predicted 12-mortality, i.e. can high end-of-life care costs be identified *ex ante*	Ensemble of RF, gradient boosting and LASSO	Administrative data: demographics, ICD codes, chronic conditions, prior utilization for a baseline sample of 5,631,168 Trajectories of health care use and diagnosis in the prior 12-month period were included	ML model attributed a higher risk score to those who died within one year than those who did not in 87% of cases End-of-life spending is high but deaths do not account heavily for high spending in Medicare overall Focusing on end-of-life spending is not a useful way to identify inappropriate treatment choices
Makar (2015) ^34^	US: Medicare fee-for- service beneficiaries in 2010	To quantify six-month mortality risk in four disease cohorts: cancer, COPD, CHF, dementia.	Six ML approaches and logistic regression were used in each cohort, of which RF models performed best in primary analysis.	Administrative data: demographics, ICD codes, chronic conditions, functional status, durable medical equipment, prior utilization for 20,000 randomly selected subjects in each disease cohort Traditional baseline characteristics were augmented with values in prior 12-month period, thus capturing disease progression, functional decline, etc.	ML model attributed a higher risk score to those who died within six months than those who did not in 82% of cases Augmented variables key to predictive power; models using only traditional variables were less accurate
Sahni (2018) ^35^	Minnesota, US: Six-hospital network (one large tertiary care centre; five community hospitals), 2012–2016	To quantify 1-year mortality risk in a cohort of clinically diverse hospitalized patients.	RF models and logistic regression were applied separately and performance compared.	Electronic medical record data, including vital signs, blood count, metabolic panel, demographics and ICD codes for 59,848 patients	ML model attributed a higher risk score to those who died within one year than those who did not in 86% of cases RF model outperforms logistic regression Demographic and lab data key to predictive power; models using ICD codes alone are less reliable

US: United States; RF: random forest; ML: machine learning; COPD: chronic obstructive pulmonary disease; CHF: congestive heart failure; ICD: international classification of disease; SEER: Surveillance, Epidemiology and End Results.

***Data synthesis*.** Narrative synthesis was employed to review the included studies and combine their key findings. Narrative synthesis involves appraisal of all relevant material, grouping the findings into a coherent thematic narrative. We chose this approach *ex ante* in the context of our broad aims: to understand how ML has been used to improve palliative care to date, and to consider the implications for future research and practice. At no point did we anticipate quantitative results for combination in meta-analysis, since both available data and ML methods would not be standardised in a way that permitted meaningful pooling.

## Results

### Database search

The database search identified 426 citations for consideration against the eligibility criteria for this review. Following removal of duplicates (n=162 *),* 264 unique citations were forwarded for title and abstract screening. Of these, 242 citations were excluded based on title and abstract, as they clearly did not meet the review’s pre-specified eligibility criteria. A full-text review of the remaining 22 citations was performed, following which a further 18 citations were excluded and one ^36^ was unobtainable. We corresponded via email with the author of the unobtainable text, who confirmed she did not have access to a copy of the manuscript and was not able to source one.

Full details for each of the 18 exclusions is provided as extended data ^32^. Reasons for exclusion at full text review were wrong methods used, e.g. not using artificial intelligence/computer learning in the study design (n=6); wrong intervention, e.g. a named drug, a stent, a surgery or chemotherapy (n=5); wrong study design, i.e. defining the sample by outcome (n=4); wrong population, i.e. under 18 (n=2); and not a peer-reviewed article (n=1). Three studies were therefore judged as eligible and included in narrative synthesis ^33– 35^; a hand-search of all references in these three papers identified no further studies of interest.

**Figure 1.**
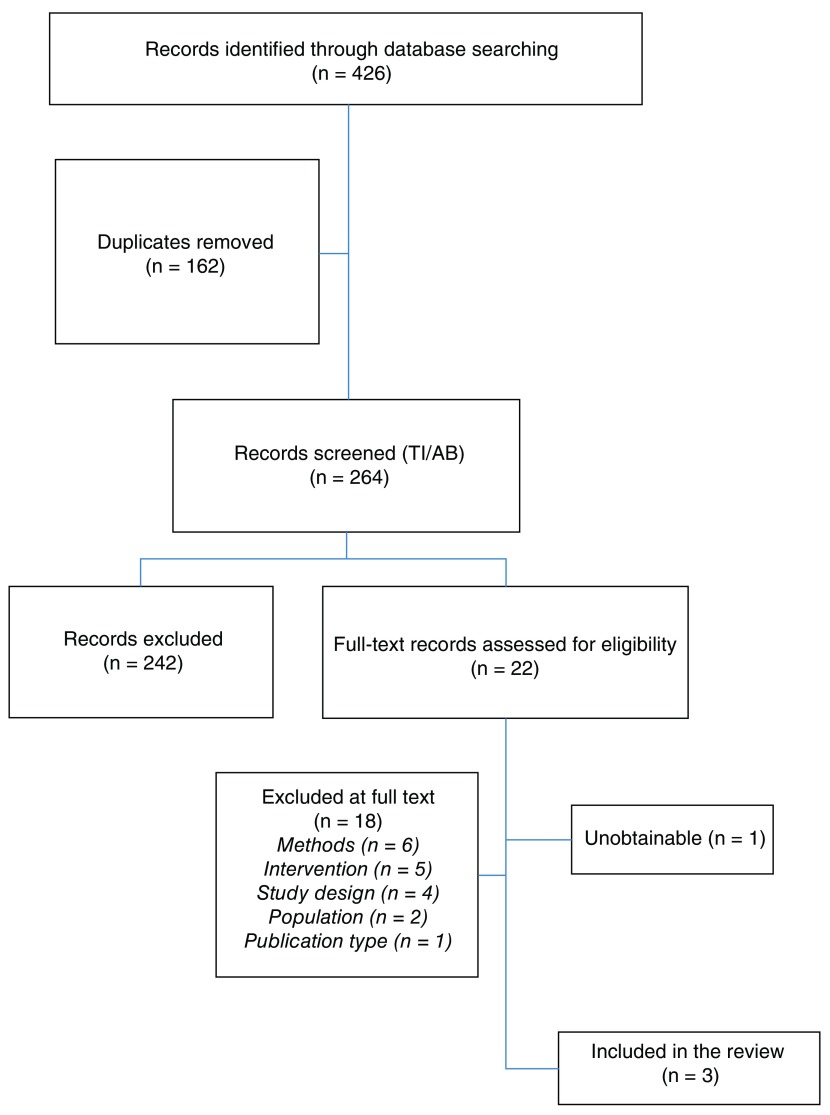
PRISMA flow diagram.

The review process is displayed in a PRISMA flow chart ( Figure 1).

### Included studies

Three studies meeting the criteria are presented in Table 1. All came from the United States, two ^33, 34^ using data from the national Medicare programme for those aged 65 and over, and one ^35^ data on adults admitted to a network of six hospitals in a single urban area. Sampling strategy and sample sizes varied: 59,848 ^35^ hospitalised adults (18+); 80,000 ^34^ Medicare beneficiaries divided equally into four disease cohorts (cancer, chronic obstructive pulmonary disease (COPD), congestive heart failure (CHF), dementia); and a representative sample of 5,631,168 ^33^ Medicare beneficiaries.

ML methods were similar: one ^35^ used random forest (RF) models only, one ^34^ used six approaches in which RF models performed best, and one ^33^ used an ensemble method combing RF, LASSO and gradient boosting approaches. All three studies used area-under-the-curve (AUC) as a measure of predictive accuracy. In this context AUC represents the probability that a randomly selected patient who died within the time horizon had a higher risk-of-mortality score than one who did not. Two ^34, 35^ studies compared the performance of their ML models to traditional logistic regression (LR) in predicting mortality.

### Main findings

In predicting 12-month mortality amongst a cohort of hospitalised adults, Sahni *et al*. report an AUC of 0.86 with ML methods versus 0.82 with logistic regression ^35^.

In predicting six-month mortality amongst disease cohorts of Medicare beneficiaries, Makar *et al.* report AUC scores of 0.83 (cancer), 0.81 (COPD), 0.76 (CHF) and 0.72 (dementia) ^34^. This performance is superior to LR using the authors’ own dataset. Sensitivity analyses suggest that unmeasured disease severity accounts for the difference in model performance across disease cohorts.

In analysing association between predicted 12-month mortality and health care expenditures in a representative sample of Medicare beneficiaries, Einav *et al.* report a weak relationship ^33^. Their AUC for 12-month mortality is 0.87 but for a population with low mortality rates (about 5% of Medicare beneficiaries died in 2008 and this proportion has since fallen), the 1% of beneficiaries with the highest mortality risk (46%<) account for less than 5% of programme expenditures. Nearly half of this “high-risk” group did not die in the 12-month time horizon. High spending is not concentrated among decedents and *ex ante* identification of mortality does not appear a useful way to identify poor value care.

### Variable selection

All papers additionally evaluate variable selection in their models.

Sahni *et al.* use a model in primary analysis that includes as predictors both routinely collected administrative data (demographics; International Classification of Disease (ICD) codes identifying presence of specific conditions) and clinical data (physiologic variables such as blood pressure, respiratory rate, body mass index; and biochemical variables such as potassium, creatinine, albumin, white blood count, platelet count). 

Age, blood urea nitrogen, platelet count, haemoglobin and creatinine were the most important predictors of 12-month mortality. Comorbidities were the least important domain for additional variables. When the model was restricted to comorbidity variables only, predictive power of the model dropped significantly (AUC=0.64) ^35^.

Makar *et al.* use as predictors routinely collected administrative data: demographics, ICD codes, chronic conditions, functional status, durable medical equipment and prior healthcare utilization. In their primary analysis, they not only employ these variables cross-sectionally at baseline but derive ‘augmented’ versions that capture trajectories, e.g. when diagnoses were made (and so how long the person is living with the condition at baseline), when functional limitations set in, when prior ED visits occurred, etc..

Augmented variables are important for model accuracy. All six models employing ML approaches lose predictive power when relying on traditional baseline variables only, e.g. the RF AUC for cancer drops to 0.77 ^34^.

Einav *et al.* employ a similar approach to Makar *et al.*, taking Medicare database variables at baseline and incorporating some prior trajectories. Additionally, they perform a sensitivity analysis with an additional variable of observed outcome, weighted to 10%. While this endogenous predictor improves the AUC to 0.96, it does not improve the model’s predictive powers for high Medicare spending ^33^.

### Model choice in the context of available variables

Sahni *et al.* and Makar *et al.* both report superior performance from RF models to traditional LR using their full datasets.

However, as noted above, RF model performance is contingent on available predictors. It is notable that when LR and RF models are compared with fewer available predictors, the superiority of RF approaches is less clear.

For Sahni *et al*., LR outperforms RF (AUC 0.71 and 0.64 respectively) when the only available clinical data are routinely collected ICD codes ^35^. For Makar *et al.*, LR performs better with only traditional variables (AUC=0.73) than with augmented (AUC=0.56). It also performs much more favourably compared to six ML approaches when only traditional variables are available.

## Discussion

### Key results

Palliative care is a field of health that may be improved through ML techniques that identify patterns and make predictions using large and complex data. A systematic search of peer-reviewed papers on this topic identifies three relevant studies with important insights.

First, ML approaches are powerful in predicting mortality in older and/or hospitalised adults. All three studies report AUC statistics that compare favourably with prior mortality prediction attempts.

Second, in reported studies these ML methods are superior to traditional LR, but only provided sufficient data are available. Particularly notable were the results of Sahni *et al*., for whom RF model performance relied on physiologic and biochemical data. When these data were excluded, LR performance was superior to RF and this performance was modest. Many studies have used comorbidity counts derived from ICD codes as predictors of important outcomes for people with serious chronic illness including short-term mortality ^37– 39^, but the papers in this review suggest these indices may not be as powerful predictors as previously understood.

Makar *et al.* similarly observe much improved RF model performance with more considered inputs, though it is notable that these were not additional clinical data but simply an inventive approach to handling routine data. The predictive power of their four models varied across disease, reflecting unmeasured underlying severity, further emphasising that input data quality decides model performance.

Third, strong predictive power does not correspond to policy implications. Einav *et al.* was the only included paper seeking to apply explicitly its mortality predictions to a policy problem (high costs near end of life), but found a strong mortality prediction model was not useful for their specific purpose. 

Corresponding implications for researchers and practitioners seeking to follow these examples are clear. Certainly, ML approaches have significant potential to improve identification of trajectories in seriously-ill populations. However, be mindful of the truism that a model is only as good as its inputs. Where possible, collect granular, individual-level clinical data. Optimise available routine data with innovative approaches where possible. Do not assume that ML approaches are *de facto* superior to traditional LR, and compare performance of different models. Perhaps most important, recognise that improving clinical decision-making will require more than simply improving the predictive power of mortality models. Expand analyses to other outcomes of interest and to the processes underpinning those outcomes.

### Limitations

This review has a number of important limitations. We conducted a rapid review of published peer review literature and not a full systematic review incorporating grey literature. Rapid reviews are a well-established methodology for gathering evidence in a limited timeframe ^30^, and we considered this appropriate for our aims of characterising the landscape of a fast-emerging field ^31^. Our results can inform ongoing attempts by researchers and practitioners to harness the power of ML methods in improving palliative care, a policy priority worldwide. A particular strength of our methods was that we retained two independent reviewers at each stage, per systematic review methodology, an approach many rapid reviews eschew ^40^. Nevertheless this is a rapidly evolving field ^16, 41^, and there are likely conference abstracts and other works in progress that would have featured in a systematic review but are excluded here.

Another limitation of the rapid review timeframe is that we did not formally assess quality or risk of bias in included papers. Instead we incorporated considerations of data quality and study usefulness in reporting, e.g. in interpreting differences in available data across studies in the context of their results. While we specified at the outset that we would not conduct a meta-analysis, we do not consider this a serious limitation in the context of our findings: specific data inputs vary across included papers yet are central to the performance of ML methods; pooling results from these papers is therefore of limited relevance.

Our eligibility criteria led to some exclusions of papers applying ML methods to improve palliative care (see extended data ^32^ for 18 papers excluded at full text review). In particular, four papers that were otherwise-eligible and examined patient outcomes, e.g. QoL, a comfortable death and related factors, were excluded for defining their sample by vital status (a sub-sample of decedents were extracted *ex post*) ^42– 45^. While decedent cohort studies have well established value in some research contexts ^12^, we established this criterion *ex ante* given endogeneity and bias concerns ^28, 29^. This decision was validated by the demonstration of one included study that found analyses of the *ex post* dead are not particularly useful in analysing *ex ante* the sick ^33^. Additionally we excluded one otherwise-eligible paper for pooling children and adults in the sample ^46^, but this decision was based in standard research practice in this field ^26^ and this was reflected in 19 of 21 (90%) full texts using adulthood as an eligibility criterion. Finally, we did not include studies where the endpoints of interest were process measures, e.g. patient-physician interaction, advance care planning and expression of patient/family preferences ^47^. While it is rational to assume that improved processes using ML will lead to improved outcomes in the long run ^16, 41^, we required that this effect on outcomes be evaluated to be eligible for our review.

One paper was excluded as unobtainable within the timeframe of the analysis, although we did subsequently obtain a copy. We did not consider the paper met our eligibility criteria. While we decided at the outset to exclude papers not in the English language, no paper was ruled ineligible on that basis.

### Future research

At the outset of this review we identified outcomes of interest in three domains: patient outcomes (survival; QoL); caregiver outcomes (survival; QoL); and economic outcomes (costs, receipt of cost-(in)effective treatment, high-intensity treatment at end of life). We specified these as established measures for quality in palliative care, and so of direct relevance to practitioners and policymakers ^27^. Most of these domains were unaddressed and so stand as priority areas for future work.

Our review included three studies with a predominant interest in patient mortality. No included study examined patient outcomes such as QoL. Evaluations of QoL are not straightforward because the outcome of interest is an individual and subjective concept where mortality is an observable binary state. Nevertheless, the reality is that living and dying with serious illness, and caring for those populations, is messy and complex. Studies characterising palliative care need beyond mortality, for example those at risk of pain or unmet need or death anxiety ^42– 45^, and accurately predicting risk would have the capacity to improve clinical decision-making and treatment pathways.

No included study, or any study rejected at full text, incorporated caregiver perspective. This is perhaps not surprising as the patient is naturally the primary focus of clinical practice and associated research. Nevertheless the role of unpaid carers is well recognised in this field, and evaluations of dyad and family outcomes is increasingly common ^48^. Identifying caregiver needs in advance would also have vast potential benefit.

One study examined an economic dimension – the long-established association between end-of-life phase and high costs. The authors conclude that this is not a useful lens by which to improve resource allocation in the care of the seriously ill. Rather, those dying with terminal illness and multimorbidity are a subset of all people living with high illness burden and high associated health care use. Identification of appropriate care and supports for this group using ML must be cognisant of recent work identifying substantive treatment effect heterogeneity by disease profile and burden in palliative care ^49^. In turn, this review has important insights for treatment effect heterogeneity work that has been based on routinely collected clinical data such as ICD codes: these data, it appears, are relatively weak predictors of relevant outcomes ^50^.

An additional concern for future studies across all outcomes of interest is timeframe. The three included studies here used six- and 12-month survival as their outcomes of interest, but there is increasing recognition that palliative care has benefits across the trajectory of life-limiting illness ^3^. For example, American Society of Clinical Oncology guidelines recommend that palliative care is provided across the disease trajectory ^51^, and the last 12 months of life accounts for less than a third of US cancer care costs ^52^. Treatment choices from diagnosis have the greatest scope to impact outcomes and costs ^53, 54^, and so studies that can inform these choices are the most useful.

Ethical issues receive scant attention in the studies read for this review. Questions arise with respect to how systems will use the data collected. For example, demographics may be important drivers of outcomes and treatment effects in the context of different experiences and preferences for care across social groups ^55, 56^. But offering or providing treatments to some groups and not others based on sociodemographic characteristics is, to say the least, ethically problematic. Also important are the data not collected. Fulfilling the goal of a “good death” involved fixed and modifiable dimensions of patient personality, experience and preferences. Personal resources associated with improved QoL near end of life may include religiosity and beliefs, ‘‘acceptance of reality’’, ‘‘life meaning and purpose’’, ‘‘self-worth’’, ‘‘hope’,’ and ‘‘caregivers’ support and acceptance’’ ^57^.

These limitations may be best addressed in the context of broad conceptual considerations for the optimal applications of big data ^58^. In all fields it is critical to ground big data collection and measurement in conceptual frameworks to guard against results that are specious, not generalisable or not actionable ^59^. This is never more true than in palliative care, where many areas of data collection and conceptual understanding are formative. No matter how powerful the artificial intelligence driving models to improve decision-making, it remains paramount that research, policy and practice protects space for the human wisdom of patients, their families and health care professionals in optimising experience of a unique life event.

## Conclusion

Improving palliative care is a policy priority worldwide. ML has the potential to support clinicians in improved decision-making by identifying those at heightened risk of inappropriate care, poor outcomes and mortality. To date studies have demonstrated capacity to improve mortality prediction. Other outcomes have not received equivalent attention. Applications of ML approaches to policy and practice remains formative. Derived results depend on available data and must be interpreted in this context. Future research must not only expand scope to consider other outcomes and longer timeframes, but also address individual needs and preferences in the context of prognosis, and engage with the profound ethical challenges of this emerging field.

## Data availability

### Underlying data

All data underlying the results are available as part of the article and no additional source data are required.

### Extended data

Open Science Framework: Appendix to: [Improving palliative care with machine learning and routine data: a rapid review]. https://doi.org/10.17605/OSF.IO/AC6TR

This project contains the following extended data:

- 20190601 Appendix.doc (Summary of search strategies; details of studies rejected at full text review) ^32^

### Reporting guidelines

Open Science Framework: PRISMA checklist and diagram for ‘Improving palliative care with machine learning and routine data: a rapid review’, https://doi.org/10.17605/OSF.IO/AC6TR ^32^

 Data are available under the terms of the Creative Commons Zero "No rights reserved" data waiver (CC0 1.0 Public domain dedication).
