# Supplementary material for: Improving palliative care with machine learning and routine data: a rapid review
Source: HRB Open Res. 2019 Aug 12;2:13. Originally published 2019 Jul 15. [Version 2] doi: 10.12688/hrbopenres.12923.2 (PMC6973530; doi:10.12688/hrbopenres.12923.2)
Supplement: Peer review history

# Peer review: Reviewer response for version 2

**DOI:** 10.21956/hrbopenres.14011.r26737

**Published:** 2019-09-16

**Authors:** Wei Gao

**Affiliations:** 1 Department of Palliative Care, Policy and Rehabilitation, Cicely Saunders Institute, King’s College London, London, UK

I think the authors have responded adequately to my concerns.

I confirm that I have read this submission and believe that I have an appropriate level of expertise to confirm that it is of an acceptable scientific standard.

# Peer review: Reviewer response for version 2

**DOI:** 10.21956/hrbopenres.14011.r26738

**Published:** 2019-08-15

**Authors:** Tim Luckett

**Affiliations:** 1 Improving Palliative, Aged and Chronic Care through Clinical Research and Translation (IMPACCT), Faculty of Health, University of Technology Sydney, Sydney, Australia

I am satisfied that the authors have addressed my previous comments.

I confirm that I have read this submission and believe that I have an appropriate level of expertise to confirm that it is of an acceptable scientific standard.

# Peer review: Reviewer response for version 2

**DOI:** 10.21956/hrbopenres.14011.r26677

**Published:** 2019-08-14

**Authors:** Amara Callistus Nwosu

**Affiliations:** 1 Palliative Care Institute Liverpool (PCIL), University of Liverpool, Liverpool, UK

The paper is well written, important and timely. The subject of machine learning and the broader topic of technology and artificial intelligence in healthcare is an emerging and important area. 

 I believe the paper should be indexed and my comments are broad to hopefully help the authors strengthen the paper.

1. The subject area is fairly narrow: Machine learning of routine data in palliative care. Consequently, few articles are included and the authors comment on the articles not included as a potential limitation. The authors do touch briefly on the reason why only routine data was chosen (i.e. the majority of BigData is routine date); however, it would be useful to have a little more justification for the choice of this (in the background) and a little more about how this may limit the broad usefulness of a paper about 'machine learning' (in the limitations), with some discussion about how analysis of other forms data with machine learning could benefit palliative care (in future research/directions).
2. Implications to policy and practice: I think a headed section of the discussion would be helpful for readers to determine whether there is evidence to change practice/policy. Also, I think it would be useful if the paper could provide a slightly clearer statement of whether the current evidence should change clinical practice or not. Currently it reads that applications to policy and practice are formative. I feel that a clear sentence stating whether clinical practice should change or not based on the evidence would be useful (e.g. 'not enough evidence to reccommend changes in policy/practice as evidence is formative etc etc).
3. Future research: It would be useful for the authors to detail the research opportunities of other forms of Big Data (e.g. wearables, apps, user generated data, non health related data (e.g. IoT devices) and other forms of artificial intelligence studies (deep learning, natural language processing).

I confirm that I have read this submission and believe that I have an appropriate level of expertise to confirm that it is of an acceptable scientific standard.

# Peer review: Reviewer response for version 1

**DOI:** 10.21956/hrbopenres.14002.r26678

**Published:** 2019-07-29

**Authors:** Wei Gao

**Affiliations:** 1 Department of Palliative Care, Policy and Rehabilitation, Cicely Saunders Institute, King’s College London, London, UK

**Major comments**

 This rapid review provides some interesting findings in a fast-emerging application area in palliative and end of life care. I enjoyed reading it. My major concern is related to the aim of this review which may also affect the choice of the search terms and the study selection.

 I think it may be more useful if this is a rapid review on current applications of ML (or even artificial intelligence) in palliative and end of life care. This might simplify the search terms and relax the inclusion criteria, thus may result in more studies (rather than 3 studies only) to be included. It is unclear why the review should be based only on those studies with some specific outcomes.

**Minor comments**

- Title may need to be changed so it reflects more accurately the aim.
- Type of intervention: care that “improves the quality of life of patients and their families facing the problems associated with a life-threatening illness”. Why “intervention” only? How about observational studies in which case “PECOTS” formula should be used? Why not other outcomes (e.g. satisfaction, functional outcomes, caregiver burden)?
- Types of studies and reports: “Studies that counted backwards, examining samples defined by characteristics at death or according to outcome, were excluded.” It needs more justifications on why these studies were excluded.

I confirm that I have read this submission and believe that I have an appropriate level of expertise to confirm that it is of an acceptable scientific standard, however I have significant reservations, as outlined above.

# Response

**Published:** 2019-08-06

**Authors:** Peter May

**Affiliations:** Trinity College Dublin, Ireland

We thank the reviewer for their comments.   These have been pasted below, numbered sequentially, and addressed as appropriate with *Response to Reviewer* and/or *Revision to Manuscript*.

We hope that these address the concerns around rationale and conclusions as returned in the original reviewer report.

**Major comments**

**2.1 This rapid review provides some interesting findings in a fast-emerging application area in palliative and end of life care. I enjoyed reading it. My major concern is related to the aim of this review which may also affect the choice of the search terms and the study selection.**

**I think it may be more useful if this is a rapid review on current applications of ML (or even artificial intelligence) in palliative and end of life care. This might simplify the search terms and relax the inclusion criteria, thus may result in more studies (rather than 3 studies only) to be included. It is unclear why the review should be based only on those studies with some specific outcomes.**

*Response to Reviewer:* As noted in the Methods>Eligiblity criteria>Type of outcomes, outcomes were defined broadly to capture those endpoints that palliative care is typically anticipated to impact. We are satisfied that this is choice of outcomes and aim is consistent with research norms in this field. As we note below in response to 2.4, the reviewer interprets incorrectly that some specific outcomes were excluded. We do not consider any relevant outcomes to have been outside our criteria and the supplementary materials show that of 18 papers read and rejected at full text, none was rejected due to choice of outcome. Also, we do not consider it feasible to revisit the aim or terms of the review once it has been completed.

*Revision to Manuscript: *None.

**Minor comments**

**2.2 Title may need to be changed so it reflects more accurately the aim.**

*Response:* Please see 2.1 response above.

**2.3 Type of intervention: care that “improves the quality of life of patients and their families facing the problems associated with a life-threatening illness”. Why “intervention” only? How about observational studies in which case “PECOTS” formula should be used?**

*Response:* Our use of the term ‘intervention’ here is consistent with the PRISMA guidelines and language (see items #4 and #6 in the PRISMA checklist).  What the PECOTS framework calls ‘exposure’, PRISMA calls ‘intervention’; in our criteria there is no material difference in use of either term, and there is no related restriction of study design.  As noted in the Methods>Eligiblity criteria>Type of studies and reports, all comparative study types including observational designs were eligible.

(Please note that in response to Reviewer 1, comment 1.8, Methods>Eligiblity criteria>Types of intervention has been merged into the preceding sub-section Types of participants)

*Revision: *None.

**2.4 Why not other outcomes (e.g. satisfaction, functional outcomes, caregiver burden)?**

*Response:* As noted in Methods>Eligiblity criteria>Type of outcomes, we specified three domains of interest: patient outcomes; caregiver outcomes; and economic outcomes.  Satisfaction and functional outcomes were both eligible outcomes under patient and caregiver outcomes, as sub-categories of quality of life.  See also 2.1 response above: no study was excluded due to specifying the “wrong outcomes”.

*Revision: *None.

**2.5 Types of studies and reports: “Studies that counted backwards, examining samples defined by characteristics at death or according to outcome, were excluded.” It needs more justifications on why these studies were excluded.** 

*Revision: *Addition to Methods>Eligibility critieria>type of studies and reports:

Studies that counted backwards, examining samples defined by characteristics at death or according to outcome, were excluded for three reasons. First, participant selection at death biases substantially derived results by distorting timeframe of analysis. Second, mortality is an outcome and many characteristics at death are outcomes; they are not independent of treatment choices but instead endogenous and therefore inappropriate as an eligibility criteria for evaluating the treatment. Third, treatment effect estimates from decedent cohort studies are in practice evidence that “treatment t should be provided to population p”, where population p is partly defined by imminent death. They are therefore only useful under scenarios of very good prognostic accuracy, and this is seldom the case in the real world.

*Response:* As well as the addition to Methods>Eligibility critieria>type of studies and reports, we also note the following lines of justification in the Discussion>Limitations, “While decedent cohort studies have well established value in some EOL research contexts, we established this criterion ex ante given endogeneity and bias concerns. This decision was validated by the demonstration of one included study that found analyses of the ex post dead are not particularly useful in analysing ex ante the sick.”

# Peer review: Reviewer response for version 1

**DOI:** 10.21956/hrbopenres.14002.r26680

**Published:** 2019-07-17

**Authors:** Tim Luckett

**Affiliations:** 1 Improving Palliative, Aged and Chronic Care through Clinical Research and Translation (IMPACCT), Faculty of Health, University of Technology Sydney, Sydney, Australia

This manuscript reports a generally well-conducted review that has been reported in accordance with PRISMA guidance. The authors call it a rapid review and seem to justify this by saying that only ’peer reviewed papers in academic journals were considered eligible’. However, I think it would be quite acceptable to call it a systematic review given that many such reviews similarly exclude grey literature on the basis of assured quality.

 Regarding terminology, I would use palliative care throughout, as it’s confusing for the reader to seemingly distinguish between these without defining the latter.

 More specific comments are as follows.

**Abstract**

- No need to repeat peer-reviewed as an eligibility criterion.
- Poor prognosis is a weak driver of costs.

**Introduction**

- The final sentence of the first paragraph is a bit harsh – I suggest replacing ‘consistently’ with ‘sometimes’.
- Palliative care is defined as an ‘interdisciplinary specialism’ but the rest of the definition is concerned with the WHO’s palliative approach to care rather than the specialist/generalist nature of the workforce delivering it. I suggest focusing just on the WHO definition.
- I am by no means an expert, but doesn’t ML only sometimes ‘make inferences without explicit user instruction' - i.e. when it is of an unsupervised kind?

**Methods**

- I found reference to ‘Types of intervention’ confusing, as this heading is usually reserved for evaluative reviews (e.g. of RCTs). Given that life-limiting illness was not mentioned under ‘Types of participants’, I suggest moving this part of the criteria under that heading. QOL is dealt with under ‘Types of outcomes’.
- Some justification is needed of excluding studies that ‘counted backwards’. This may also need further explanation in the context of the predominance of AUC, which seems to focus on people who died and then look back to see what predicted this.
- The English language requirement needs to be included under eligibility rather than Information sources.
- The Information sources heading needs to also include ‘Search’.
- ‘Hand searching’ is a more widely used term than ‘Snowball sampling’ for reviews.
- Remove the heading ‘Data analysis’ and leave in those for extraction and synthesis.

**Findings**

- Unless I have misunderstood, this section seems to refer to other studies that were not included in the review and so should be moved to the Discussion. But what is meant by ‘prior mortality prediction efforts’ and which authors are referred to in ‘the authors’ own dataset’? If the authors of the paper in question, then it should be written in the past tense.
- I lack expertise to comment on interpretations brought to the different statistical approaches used in the studies.

**Discussion**

- Here, ‘prior mortality prediction attempts’ really is presumably referring to other research that does not use ML; references are needed.
- I don’t quite follow the statement ‘This places a question mark against the assumption in much palliative care literature that comorbidity counts derived from ICD codes are important predictors of short-term mortality’, and again references are needed.
- The justification that quality appraisal was outside the authors’ ‘sphere of interest’ reads oddly and is not a normally accepted justification. Could it be, instead, that existing quality appraisal tools are not a good fit for studies using routine data?
- I recommend removing reference to meta-analysis in the limitations, as this is totally unsuited to the review’s aims.
- I wonder if reference to some other of the issues raised in the following article might be pertinent to the Discussion ^1^.

I confirm that I have read this submission and believe that I have an appropriate level of expertise to confirm that it is of an acceptable scientific standard, however I have significant reservations, as outlined above.

## References

[1] : Hope, hype and harms of Big Data. *Intern Med J*.2019;49(1) : 10.1111/imj.14172 126-129 10.1111/imj.14172 30680901

# Response

**Published:** 2019-08-06

**Authors:** Peter May

**Affiliations:** Trinity College Dublin, Ireland

We thank the reviewer for their comments.   These have been pasted below, numbered sequentially, and addressed as appropriate with *Response to Reviewer* and/or *Revision to Manuscript*.

**GENERAL COMMENTS**

**1.1 This manuscript reports a generally well-conducted review that has been reported in accordance with PRISMA guidance. The authors call it a rapid review and seem to justify this by saying that only ’peer reviewed papers in academic journals were considered eligible’. However, I think it would be quite acceptable to call it a systematic review given that many such reviews similarly exclude grey literature on the basis of assured quality.** 

*Response to Reviewer*: We appreciate this comment. There is an obvious incentive to make this change as an SR ranks higher than an RR. However it feels a little intellectually disingenuous to have defined this as an RR ourselves, conducted the whole thing to a tight time frame (circa 6 months) and excised various elements of an SR that I would consider non-negotiable (grey lit, PROSPERO registration). Despite the temptation then, I’m minded to leave as is unless instructed otherwise by editors and reviewers.

*Revision to Manuscript*: None.

**1.2 Regarding terminology, I would use palliative care throughout, as it’s confusing for the reader to seemingly distinguish between these without defining the latter.**

*Revision*: ‘Palliative and EOL care’ as a sphere of interest has been changed throughout to ‘palliative care’, including the paper title. A small handful of ‘end of life’ uses remain where appropriate to specific context.

**Abstract**

**1.3 No need to repeat peer-reviewed as an eligibility criterion.**

*Revision*: Final clause of the Methods deleted.

**1.4 Poor prognosis is a weak driver of costs.**

*Revision*: Not clear exactly what this comment implies but perhaps it’s a poor explanation of the finding. 

Revised final two sentences of the Results:

ML-informed models outperformed logistic regression in predicting mortality where data inputs were relatively strong, but models using only routine administrative data had limited benefit from ML methods. Identifying poor prognosis does not appear effective in tackling high costs associated with serious illness. 

**Introduction**

**1.5 The final sentence of the first paragraph is a bit harsh – I suggest replacing ‘consistently’ with ‘sometimes’.**

*Revision*: Changed to ‘often’.

**1.6** **Palliative care is defined as an ‘interdisciplinary specialism’ but the rest of the definition is concerned with the WHO’s palliative approach to care rather than the specialist/generalist nature of the workforce delivering it. I suggest focusing just on the WHO definition.** 

*Revision*: Reference to interdisciplinary specialism removed.

**1.7** **I am by no means an expert, but doesn’t ML only sometimes ‘make inferences without explicit user instruction' - i.e. when it is of an unsupervised kind?** 

*Revision*: **can** make inferences without explicit user instruction.

**Methods**

**1.8 I found reference to ‘Types of intervention’ confusing, as this heading is usually reserved for evaluative reviews (e.g. of RCTs). Given that life-limiting illness was not mentioned under ‘Types of participants’, I suggest moving this part of the criteria under that heading. QOL is dealt with under ‘Types of outcomes’.**

*Revision*: ‘Type of intervention’ section merged into ‘Type of particpants’.

**1.9** **Some justification is needed of excluding studies that ‘counted backwards’. This may also need further explanation in the context of the predominance of AUC, which seems to focus on people who died and then look back to see what predicted this.**

*Revision*: Addition to Methods>Eligibility critieria>type of studies and reports (also in response to Reviewer 2, comment 2.5):

Studies that counted backwards, examining samples defined by characteristics at death or according to outcome, were excluded for three reasons. First, participant selection at death biases substantially derived results by distorting timeframe of analysis. Second, mortality is an outcome and many characteristics at death are outcomes; they are not independent of treatment choices but instead endogenous and therefore inappropriate as an eligibility criteria for evaluating the treatment. Third, treatment effect estimates from decedent cohort studies are in practice evidence that “treatment t should be provided to population p”, where p is partly defined by imminent death. They are therefore only useful under scenarios of very good prognostic accuracy, and this is seldom the case in the real world.

*Response*: Studies using AUC can either do prospectively (enrolling people at baseline and then following them until death and then using baseline data to create and validate a mortality-prediction index) or retrospectively (scooping up all data after the fact then using baseline data to create and validate a mortality-prediction index), and included studies do this. No included studies (but a number of excluded full texts in the supplementary materials) defined the sample at death and counted backwards where any baseline is created ex post by the investigator, which is a recipe for bias ( https://www.ncbi.nlm.nih.gov/pubmed/15585737).

**1.10** **The English language requirement needs to be included under eligibility rather than Information sources.**

*Revision:* Moved to end of ‘types of studies and reports’.

**1.11 The Information sources heading needs to also include ‘Search’**

*Revision:* Added as Table 1.

**1.12** **‘Hand searching’ is a more widely used term than ‘Snowball sampling’ for reviews.**

*Revision:* Changed to ‘Hand searching’.

**1.13** **Remove the heading ‘Data analysis’ and leave in those for extraction and synthesis.#**

*Revision:* Deleted heading.

**Findings**

**1.14 Unless I have misunderstood, this section seems to refer to other studies that were not included in the review and so should be moved to the Discussion. But what is meant by ‘prior mortality prediction efforts’ and which authors are referred to in ‘the authors’ own dataset’? If the authors of the paper in question, then it should be written in the past tense.**

*Revision:* Reference to papers from outside the review deleted in Results (and referenced in Discussion, see 1.16).

**1.15 I lack expertise to comment on interpretations brought to the different statistical approaches used in the studies.**

*Revision:* None.

**Discussion**

**1.16 Here, ‘prior mortality prediction attempts’ really is presumably referring to other research that does not use ML; references are needed.** 

*Revision:* Relevant systematic review referenced.

**1.17** **I don’t quite follow the statement ‘This places a question mark against the assumption in much palliative care literature that comorbidity counts derived from ICD codes are important predictors of short-term mortality’, and again references are needed.**

*Revision:* Sentence revised as: Many studies have used comorbidity counts derived from ICD codes as predictors of important outcomes for people with serious chronic illness including short-term mortality (refs), but the papers in this review suggest these indices may not be as powerful predictors as previously understood.

**1.18 The justification that quality appraisal was outside the authors’ ‘sphere of interest’ reads oddly and is not a normally accepted justification. Could it be, instead, that existing quality appraisal tools are not a good fit for studies using routine data?**

*Revision:* I’ve deleted this sentence as it’s not needed or useful.

**1.19 I recommend removing reference to meta-analysis in the limitations, as this is totally unsuited to the review’s aims.**

*Response:* I tend to agree but a colleague who reviewed a draft of this pre-submission was insistent that it was relevant. So I prefer to include to be clear that we considered it and don’t think it’s relevant (even though some readers may do so).

**1.20 I wonder if reference to some other of the issues raised in the following article might be pertinent to the Discussion.**

*Revision: *Final paragraph before the conclusion is extended:

These limitations may be best addressed in the context of broad conceptual considerations for the optimal applications of big data.  In all fields it is critical to ground big data collection and measurement in conceptual frameworks to guard against results that are specious, not generalisable or not actionable.  This is never more true than in palliative care, where many areas of data collection and conceptual understanding are formative.
